# Increased plasma sVCAM-1 is associated with severity in IgA nephropathy

**DOI:** 10.1186/1471-2369-14-21

**Published:** 2013-01-22

**Authors:** Li Zhu, Sufang Shi, Lijun Liu, Jicheng Lv, Hong Zhang

**Affiliations:** 1 Renal Division, Department of Medicine, Peking University First Hospital; Peking University Institute of Nephrology; Key Laboratory of Renal Disease, Ministry of Health of China; Key Laboratory of Chronic Kidney Disease Prevention and Treatment (Peking University), Ministry of Education, Beijing, 100034, China

**Keywords:** sVCAM-1, IgA nephropathy, Vascular injury

## Abstract

**Background:**

A considerable proportion of IgAN patients present with histological vasculitic/crescentic lesions in glomeruli, indicating activation of vascular inflammation. Using sVCAM-1, a well-proven marker for endothelial injury under inflammatory processes, we investigated vascular injury and its association with clinical and pathological manifestations in IgAN patients.

**Methods:**

In this study, 327 biopsy-proven IgAN patients and 55 healthy controls were enrolled. The Oxford classification and two variables, Active Crescentic Lesion Percentage (ACLP) and Chronic Glomerular Lesion Percentage (CGLP), were used for evaluating pathological lesions. Human Umbilical Vein Endothelial Cells were treated with 25-400 ug/ml IgA1. sVCAM-1 in plasma and culture supernatant were measured by ELISA.

**Results:**

Plasma sVCAM-1 in IgAN patients was significantly higher than healthy controls. In patients with IgAN, plasma sVCAM-1 was significantly correlated with eGFR, 24h urine protein excretion, tubular atrophy/interstitial fibrosis lesion and ACLP, but not CGLP. Meanwhile, compared to healthy volunteers, IgA1 from IgAN patients showed a tendency to increase the HUVECs supernatant sVCAM-1 expression. And IgA1 induced the sVCAM-1 increasing from HUVECs in time- and dose-dependent manner.

**Conclusions:**

We found increased plasma sVCAM-1 in IgAN patients and its association with severe clinical and pathological manifestations, which might be partly resulted from effect of IgA1 to endothelial cells.

## Background

IgA nephropathy (IgAN) is the most common primary glomerulonephritis worldwide [1]. Except for the most characteristic finding, that is glomerular mesangial area deposition of IgA, a considerable proportion of IgAN patients present with histological vasculitic/crescentic lesions in glomeruli, accompanied by distinct neutrophils and/or monocytes infiltration, indicating the activation of vascular inflammation in IgAN [2]. Clinically, many patients with IgAN suffer hypertension at a relative young age, and some present with secondary malignant hypertension. In China, IgAN was reported as one of the major causes of secondary malignant hypertension, accounting for one-third of all secondary causes and 40% of glomerular causes [3,4]. Although clinical and histological observations strongly indicated the involvement of vascular injury in IgAN, the pathogenesis of the vascular injury in IgAN is still unknown.

Vascular cell adhesion molecule 1 (VCAM-1) is endothelial ligand for integrins expressed on leukocytes and platelets, with the function of facilitation endothelial adhesion of circulating leukocytes. The endothelial expression of VCAM-1 is increased in response to inflammatory cytokines [5]. And the soluble ectodomain of VCAM-1 (sVCAM-1) is proteolytically released from the endothelial cell surface into the circulation upon endothelial activation and injury. Elevated plasma sVCAM-1 level was reported in many diseases, such as coronary and peripheral atherosclerosis, diabetes mellitus, systemic lupus erythematosus, Crohn's disease and so on [6-9]. Soluble vascular adhesion molecule-1 is now a well-proven marker for endothelial injury under inflammatory processes.

In patients with chronic kidney disease (CKD), including IgAN, elevated plasma sVCAM-1 level was also observed and reported to be associated with impaired renal function [10-13]. But most studies were unable to state the major cause of high plasma levels of sVCAM-1, which could be either explained by increased production or decreased clearance. Recently, A.E.M. Stinghen et al. reported that exposure of endothelial cells to uremic plasma resulted in a time- and CKD-stage-dependent increased expression of sVCAM-1, suggesting a link between vascular activation, systemic inflammation and uremic toxicity [14]. IgA nephropathy, a study is needed to explore the cause of high plasma levels of sVCAM-1 and its association with vascular inflammation.

In the present study, we investigated the association of sVCAM-1 with clinical and pathological characteristics in patients with IgAN, and further detected the effect of IgA1 molecules on sVCAM-1 production by cultured endothelial cells in vitro to explore the mechanism of vascular inflammation in IgAN.

## Methods

### Study population

In the present study, a total of 382 subjects were enrolled, including 327 IgAN patients and 55 healthy volunteers with normal urine analysis and blood pressure. Diagnoses of IgAN were biopsy proven and confirmed by granular deposition of IgA in the glomerular mesangium by immunofluorescence detection, as well as by the deposition of electron dense material in mesangial ultra-structural examination. Patients with Henoch-Schonlein purpura, systemic lupus erythematosus, and chronic hepatic diseases were excluded by detailed clinical and laboratory examinations. In addition, 19 patients with minimal change disease (MCD), 30 with membranous nephropathy (MN) and 25 with lupus nephritis were recruited as control patients with other glomerular injury.

On the morning of renal biopsy and the inclusion in the study, plasma (EDTA) from patients and healthy volunteers was collected, divided into aliquots, and stored at −80°C pending the measurement of sVCAM-1. Clinical manifestations at the time of renal biopsy, including serum IgA level, serum creatinine level, 24 hours urine protein excretion, history of hypertension and history of gross hematuria were collected from the clinical record. The glomerular filtration rate (GFR) of IgAN patients was calculated by the Modified Glomerular Filtration Rate Estimating Equation for Chinese [15]. Histologically, the Oxford classification was used for evaluating the pathological lesions [16]. Additionally, we used two indices to grossly distinguish the active and chronic glomerular lesion, which were Active Crescentic Lesion Percentage (ACLP) and Chronic Glomerular Lesion Percentage (CGLP). ACLP included the glomerular bearing cellular crescents, fibrocellular crescents and fibrinoid necrosis. CGLP included the glomerular having global sclerosis, segmental sclerosis and fibrous crescents.

The protocol for this study was approved by the Medical Ethics Committee of Peking University and informed written consent for this study was obtained from every participant.

### Detection of plasma sVCAM-1

Plasma sVCAM-1 levels were determined using commercial ELISA kits according to the manufacturer’s specifications (R&D Systems, Minneapolis, MN, USA).

### Isolation of human plasma IgA1

Whole IgA1 was isolated by jacalin affinity chromatography from plasma as previous described [17]. In the present study, another small population, including 12 biopsy approved IgAN patients and 12 healthy controls, was used for IgA1 isolation. Plasma from the 24 individuals was introduced to jacalin affinity chromatography, one by one. Briefly, 3ml plasma from every subject were diluted 1:3 with 0·01 mol/l phosphate buffered saline (PBS), filtered through a 0.22 um Corning syringe filter (Corning Glass Works, Corning, NY, USA) and then applied to a jacalin column, which was prepared with commercially available jacalin immobilized on cross-linked 4% beaded agarose (Pierce Chemical Company, Rockford, IL, USA) with an IgA1 binding capacity of more than 2 mg/ml. The column was equilibrated with 0.01 mol/l PBS and IgA1 was eluted with 0.1 mol/l melibiose (Sigma, St. Louis, MO, USA) in 0.01 mol/l PBS. The eluted sample concentration and removal of melibiose were performed by pressure ultrafiltration using Vivaspin (Cat No: VS2021, Sartorius, Goettingen, Germany). The total protein concentrations of isolated IgA1 samples from the 24 individuals were tested using *DC* Protein Assay (Bio-Rad, Hercules, CA, USA). IgA1 was then aliquoted and stored at −80°C pending cell culture experiments.

### Culture of endothelial cells with IgA1

Primary Human Umbilical Vein Endothelial Cells (HUVECs) and all the cell culture media, fetal bovine serum, supplements and extra cellular matrix were purchased from ScienCell Corporation (ScienCell, Carlsbad, CA, USA). Cells were cultured according to the manufacturer’s specifications, in Endothelial Cell Medium (ECM) supplemented with Endothelial Cell Growth Supplement (EsCGS), 5% fetal bovine serum, penicillin G (100 U/ml) and streptomycin (100 U/ml) at 37°C in a humidified 5% CO_2_ incubator. HUVECs were seeded into 24-well plates precoated with 1 ug/cm^2^ Poly-L-Lysine (PLL) before experiment. After 12 h of serum starving, HUVECs were treated with 400 ug/ml whole IgA1 from 24 individuals, including 12 IgAN patients and 12 healthy controls (we did not pool isolated IgA1 from different IgAN patients and healthy controls together). For dose-dependent assay, 25-400 ug/ml whole IgA1 from one IgAN patient were used. For time-dependent assay, HUVECs were treated with 200 ug/ml whole IgA1 from one IgAN patient for 6–48 hours.

### Detection of supernatant sVCAM-1

Supernatant samples were separated from culture after 48h of incubation (except for time-dependent assay, in which 6-48 h were used) and were stored at −80°C until assay. Supernatant sVCAM-1levels were measured by ELISA as described above.

### Statistical analysis

Descriptive statistical analyses were performed with SPSS10.0 software (SPSS Inc., USA). Continuous variables were compared by unpaired Student’s t test or ANOVA (ANalysis Of VAriance between groups). Dichotomous and polychromous data were analyzed by the χ2 test. Correlations were performed by the Spearman rank test (ρ). Results are expressed as means ± SD. A p value of less than 0.05 was considered statistically significant.

## Results

### Clinical and pathological manifestations of patients with IgAN

In our study, 327 IgAN patients were involved. The main clinical and pathological manifestations of them were summarized in Table 1. Of them, 155 were males and 172 were females. The age at renal biopsy was 32.9±10.7 year old. And 30.9% patients were with a history of macroscopic hematuria, 37.9% with a history of hypertension and 64.5% with moderate and severe proteinuria (>1 g/24 h). eGFR were 85.43±30.29 ml/min.

**Table 1:** Clinical and pathological manifestations of enrolled IgAN patients

**Variables**			**Manifestations**
Patients number			327
age (y)			32.9±10.7
eGFR (ml/min)			85.43±30.29
gender	Male		155
	Female		172
macroscopic hematuria	with		101 (30.9%)
	without		226 (69.1%)
proteinuria	more than 1 g/24 h		211 (64.5%)
	less than 1 g/24 h		116 (35.5%)
hypertension	with		124 (37.9%)
	without		203 (62.1%)
Oxford classification	M	M0	123 (37.6%)
		M1	204 (62.4%)
	E	E0	141 (43.1%)
		E1	186 (56.9%)
	S	S0	68 (20.8%)
		S1	259 (79.2%)
	T	T0	253 (77.4%)
		T1	39 (11.9%)
		T2	35 (10.7%)

### Plasma levels of sVCAM-1 in the IgAN patients

The plasma levels of sVCAM-1 did not significantly differ between males and females in IgAN patients. Patients with IgAN presented higher plasma levels of sVCAM-1 than that in healthy controls (726.66±290.35 ng/ml Vs 520.23±137.51 ng/ml, p<0.001). In patients with MCD and MN, plasma levels of sVCAM-1 showed no difference to healthy controls (MCD: 485.09±203.37 ng/ml, MN: 511.49±278.36 ng/ml), while patients with LN presented higher levels of sVCAM-1 (LN: 958.53±601.49, p value versus healthy controls=0.001), which was similar to that found in IgAN patients.

### Correlations between plasma levels of sVCAM-1 and clinical characteristics of patients with IgAN

**Figure 1:**
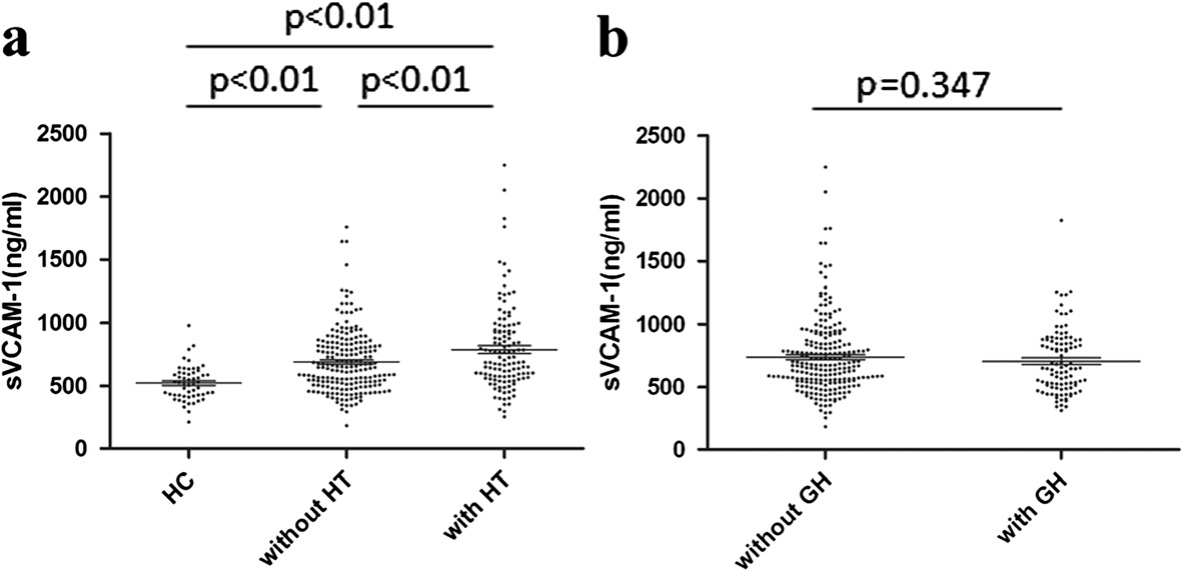
**Scatter plot showing distribution of plasma sVCAM-1 levels of enrolled individuals according to with or without hypertension (HT), and with or without gross hematuria history (GH), respectively.a**. Both group of IgAN patients, with or without hypertension, presented with higher plasma sVCAM-1 levels than healthy controls (without hypertension Vs healthy controls: 690.17±249.75 ng/ml Vs 520.23±137.51 ng/ml, p<0.01; with hypertension Vs healthy controls: 786.40±339.43 ng/ml Vs 520.23±137.51 ng/ml, p<0.01). IgAN patients with hypertension had significantly elevated plasma sVCAM-1 levels than those without (786.40±339.43 ng/ml Vs 690.17±249.75 ng/ml, p<0.01).**b.** Plasma levels of sVCAM-1 showed no difference between IgAN patients with or without gross hematuria history.

In patients with IgAN, the plasma levels of sVCAM-1 were significantly correlated with eGFR (ρ=−0.197, p<0.001), 24 h urinary protein excretion (UPE) (ρ=0.198, p<0.001) and uric acid (UA) (ρ=0.117, p=0.038) (Table 2), but not serum IgA levels, systolic blood pressure and diastolic blood pressure at the time of renal biopsy. Although the blood pressure at the time of renal biopsy did not correlated with plasma sVCAM-1 levels, IgAN patients with hypertension still had significantly elevated plasma sVCAM-1 levels than those without (786.40±339.43 ng/ml Vs 690.17±249.75 ng/ml, p<0.001) (Figure 1a). Both group of patients, with or without hypertension, presented with higher plasma sVCAM-1 levels than healthy controls (without hypertension Vs healthy controls: 690.17±249.75 ng/ml Vs 520.23±137.51 ng/ml, p<0.01; with hypertension Vs healthy controls: 786.40±339.43 ng/ml Vs 520.23±137.51 ng/ml, p<0.01) (Figure 1a). The plasma levels of sVCAM-1 showed no difference between IgAN patients with or without gross hematuria history (Figure 1b).

**Table 2:** Correlations between plasma levels of sVCAM-1 and clinical and histological manifestations of patients with IgAN

	**eGFR**	**UPE**	**IgA**	**UA**	**SBP**	**DBP**	**ACLP**	**CGLP**
ρ*	−0.197	0.198	0.062	0.117	0.077	0.053	0.182	0.054
p value	<0.001	<0.001	0.293	0.038	0.168	0.344	0.001	0.327

* Pearson's correlation coefficient.

### Correlations between plasma levels of sVCAM-1 and pathological characteristics of patients with IgAN

Patients with higher scores in the variable tubular atrophy/interstitial fibrosis showed significantly higher sVCAM-1 (T2:T1:T0=941.97±339.95 ng/ml: 699.27±213.43 ng/ml:701.10±281.76 ng/ml, p<0.001), patients with higher scores in the variables mesangial hypercellularity showed relative higher sVCAM-1, although do not reach the statistic significance (M1:M0=749.88±279.76 ng/ml: 688.14±304.38 ng/ml, p=0.062) (Table 3). Furthermore, the plasma levels of sVCAM-1 significantly correlated with Active Crescentic Lesion Percentage (ACLP), but not Chronic Glomerular Lesion Percentage (CGLP) (Table 2).

**Table 3:** Plasma sVCAM-1 levels of IgAN patients with different histological lesions, according to Oxford classification

** Oxford classification**		**Number**	**sVCAM-1 (ng/ml)**	**p value**
M	0	123	688.14±304.38	0.062
	1	204	749.88±279.76	
E	0	141	716.72±276.41	0.591
	1	186	734.20±301.01	
S	0	68	755.98±389.63	0.46
	1	259	718.96±258.49	
T	0	253	701.10±281.76	<0.001
	1	39	699.27±213.43	
	2	35	941.97±339.95	

### sVCAM-1 expression induced by IgA1 in HUVECs

**Figure 2:**
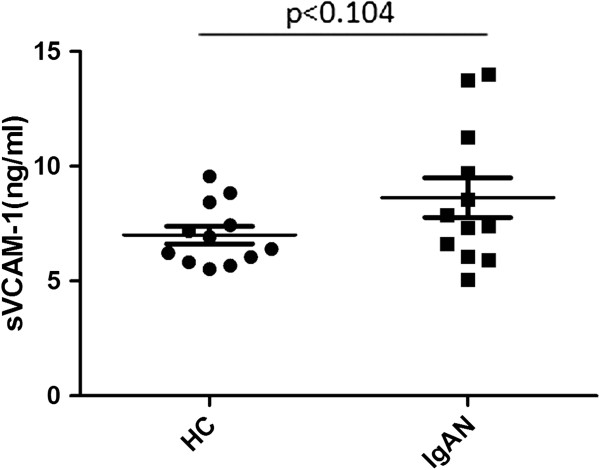
**Scatter plot showing sVCAM-1 expressions in cultured HUVECs under IgA1 from IgAN patients and healthy volunteers.** HUVECs were treated with 400 ug/ml IgA1 from healthy volunteers (HC) or IgAN patients (IgAN). Compared to IgA1 from healthy volunteers, IgA1 form IgAN patients showed a tendency to increase the HUVECs supernatant sVCAM-1 expression (8.63±2.98 ng/ml Vs 7.00±1.33 ng/ml, p=0.104).

**Figure 3:**
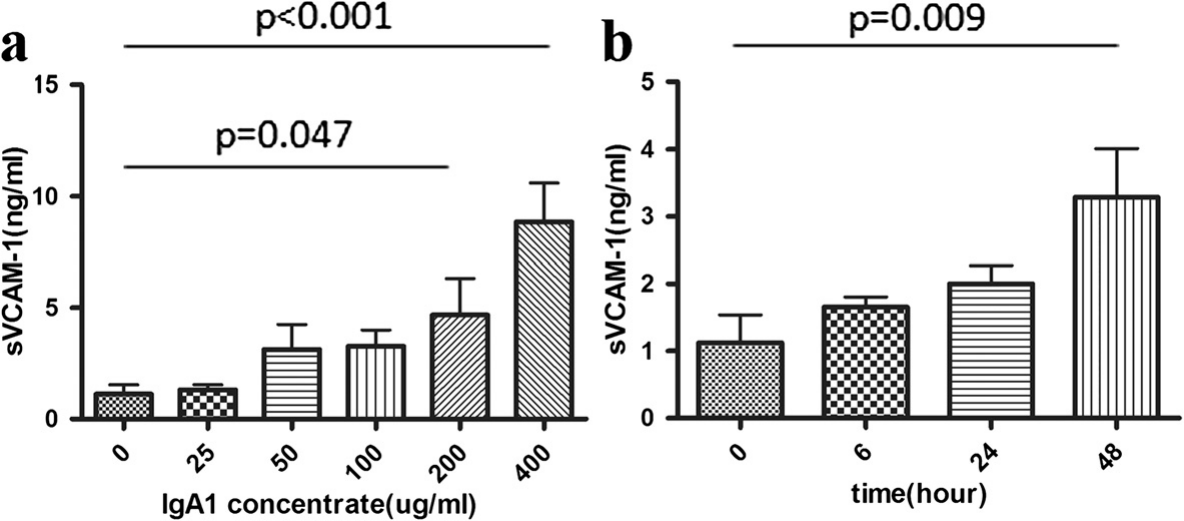
**sVCAM-1 expressions in cultured HUVECs under different doses of IgA1 treatment for different time durations.a**. HUVECs were treated with 25-400 ug/ml IgA1 for 48 hours. IgA1 induced the sVCAM-1 expression in cultured HUVECs supernatant in a dose-dependent manner. Significantly increased supernatant sVCAM-1 level could be detected at 200 ug/ml and 400 ug/ml IgA1 (p=0.047 for 200 ug/ml, p<0.001 for 400 ug/ml).**b**. HUVECs were treated with 200 ug/ml IgA1 for 6–48 hours. IgA1 induced the sVCAM-1 expression in cultured HUVECs supernatant in a time-dependent manner. The sVCAM-1 level in HUVECs supernatant significantly elevated upon 48 h treatment (p=0.009).

At first, using HUVEC as a model of endothelial cells, we tested the sVCAM-1 induction effect by IgA1 from 24 different subjects, which were 12 IgAN patients and 12 healthy volunteers. Compared to IgA1 from healthy volunteers, IgA1 form IgAN patients showed a tendency to increase the HUVECs supernatant sVCAM-1 expression, although did not reach the statistic significance (Figure 2). Next, we detected the effect of IgA1 on the sVCAM-1 expression in vitro. We found that IgA1 increased the sVCAM-1 expression in HUVECs culture supernatant in a time- and dose-dependent manner. The sVCAM-1 level in HUVECs supernatant significantly elevated upon 48 h treatment with 200-400 ug/ml IgA1 (Figure 3a). And significantly increased supernatant sVCAM-1 level could be detected at 48 h (Figure 3b).

## Discussion

Patients with IgA nephropathy presented with variable clinical findings, histological impairments and long-term renal outcomes [18]. In the present study, we enrolled 327 patients with IgAN. Among them, 37.9% patients presented with clinical hypertension at the time of renal biopsy, which was well-approved to be an independent risk factor for long-term renal outcome [19,20]; and 56.9% patients presented with histological endocapillary hypercellularity, according to Oxford classification. This supported previous observations that a great mass of IgAN patients suffered vascular injury [21]. When we used sVCAM-1 as the marker for vascular injury, especially inflammation injury, results showed that patients with IgAN had significantly elevated plasma sVCAM-1 compared to healthy controls, which was in accordance with previous reports in other IgAN patients’ cohorts [11]. Similarly, in patients with lupus nephritis, in which condition vascular injury were commonly reported, sVCAM-1 levels were also significantly higher. But in some other glomerular injury conditions, such as MCD and MN, patients showed comparable sVCAM-1 levels to healthy controls. Therefore, we regarded sVCAM-1 as an indicator to vascular injury other than glomerular injury.

Hypertension is a well accepted clinical finding for vascular injury, but still over 70% patients in our present study did not have this obvious symptom for vascular injury. Although without hypertension, they also showed significantly higher plasma sVCAM-1 compared to healthy controls, while the patients with hypertension presented with even higher plasma sVCAM-1. From above findings, we suggested that plasma sVCAM-1 as another marker for vascular injury, except for clinical hypertension, and IgAN patients suffered vascular injury are likely underestimated.

In addition, we described the significant association between plasma sVCAM-1 level and clinical findings, including eGFR and urinary protein excretion. D’Amico G has already came to the concordance that impairment of renal function, severe proteinuria, and arterial hypertension were the strongest and more reliable clinical predictors of an unfavorable outcome after critical analysis of results of 30 studies [22]. Since plasma sVCAM-1 level in patients with IgAN associated with all the three risk factors, we speculated the patients had higher plasma sVCAM-1 levels would be in more severe clinical states of IgAN.

Accordingly, we found the significantly different plasma sVCAM-1 level among patients with different tubular atrophy/interstitial fibrosis lesion judged by Oxford classification, an independent predictor of long-term renal outcome measured by multivariate analysis. This result indicated that plasma sVCAM-1 level in patients was a marker not only for severe clinical state but also for severe histological lesion of IgAN.

Oxford classification was derived from a group of IgAN patients without most acute and rapidly progressive disease courses and more than 50% of them without crescent, but many of our IgAN patients in the present study had crescentic lesion in the glomeruli. Therefore, we used two variables, Active Crescentic Lesion Percentage (ACLP) and Chronic Glomerular Lesion Percentage (CGLP), to roughly distinguish the acute and chronic histological lesions. We surprisingly found that only ACLP was significantly associated with plasma sVCAM-1 level, not CGLP. It is important for us to point out that plasma sVCAM-1 level was a marker for acute crescentic lesion in patients with IgAN. Crescentic lesion is actually a kind of glomerular vascular inflammation, so we considered elevated plasma sVCAM-1 level in patients with IgAN can at least partly ascribed to glomerular vascular inflammation lesion.

Furthermore, we wanted to explore the reason of the elevated sVCAM-1 in IgAN. In recent years, lots of efforts have been made to uncover the pathogenesis of IgAN, and some significant progress has been achieved in this field [23-25]. Increasing evidences indicated abnormal IgA1 molecules from IgAN patients to be the initial factor to induce glomerular mesangial cells proliferation and secondary podocytes and tubular epithelial cells injury [26-29]. Regarding to endothelial cells, less evidence was available. We hypothesized that abnormal IgA1 molecules from IgAN patients had unique activity to promote sVCAM-1 expression from endothelial cells. In order to exclude other reasons induced sVCAM-1 elevation in IgAN patients, such as inflammation induced increased production by inflammatory cells and eGFR induced decreased clearance, an in vitro cell model was used. In the present study, we inferred that IgA1 molecules from IgAN patients could promote sVCAM-1 expression of endothelial cells for the following three evidences: (1) the same amount of IgA1 from IgAN patients showed a tendency to increase endothelial sVCAM-1 expression; (2) patients with IgAN presented with higher levels of IgA1 compared to healthy controls; (3) IgA1 induced the sVCAM-1 increasing from HUVECs in dose-dependent manner. However, it was very possible that many other factors, like inflammatory cells activation and decreased clearance due to impaired renal function were also contributed to elevated sVCAM-1 in IgAN patients, which we could not determine from present study.

## Conclusions

In the present study, we used sVCAM-1 as a marker to investigate vascular injury and its association with clinical and pathological characteristics in patients with IgAN. We found that the plasma levels of sVCAM-1 in patients with IgAN were increased. And increased plasma sVCAM-1 was associated with severe clinical and pathological findings, especially acute glomerular vascular inflammation lesion in patients with IgAN, which might be partly resulted from the effect of IgA1 to endothelial cells.

## Abbreviations

IgAN: IgA nephropathy; sVCAM-1: Soluble Vascular cell adhesion molecule 1; CKD: Chronic kidney disease; eGFR: Estimated glomerular filtration rate; ACLP: Active Crescentic Lesion Percentage; CGLP: Chronic Glomerular Lesion Percentage; HUVECs: Human Umbilical Vein Endothelial Cells.

## Competing interests

The authors have declared that no competing interests exist.

## Authors’ contributions

LZ participated in the design of the study, carried out the molecular biological and cell biological studies and drafted the manuscript. SS carried out the pathological classification. LL recruited patients and participated in the ELISA detection. JL recruited patients and performed the statistical analysis. HZ conceived of the study, and participated in its design and coordination and helped to draft the manuscript. All authors read and approved the final manuscript.

## Pre-publication history

The pre-publication history for this paper can be accessed here:

http://www.biomedcentral.com/1471-2369/14/21/prepub
